# PD-1 or PD-L1 inhibitors in addition to first-line chemotherapy for endometrial cancer: an extracted individual patient data meta-analysis

**DOI:** 10.3332/ecancer.2025.1884

**Published:** 2025-04-01

**Authors:** Mariana Carvalho Gouveia, Renata Colombo Bonadio, Felippe Lazar Neto, Maísa Maria Spagnol Trento, Mateus Trinconi Cunha, Mariana Scaranti

**Affiliations:** 1 Hospital Nove de Julho – DASA Oncologia, São Paulo, Brazil; 2 Instituto do Câncer do Estado de São Paulo, University of São Paulo, São Paulo, Brazil; 3 Instituto D’Or de Pesquisa e Ensino (IDOR), São Paulo, Brazil; 4 Sunnybrook Health Sciences Centre, University of Toronto, Toronto, Canada; a https://orcid.org/0000-0001-8194-7594; b https://orcid.org/0000-0001-5818-922X; c https://orcid.org/0000-0002-0051-9537; d https://orcid.org/0000-0002-9436-2146; e https://orcid.org/0000-0002-9436-2146; f https://orcid.org/0000-0001-7713-0100; † Mariana Carvalho Gouveia and Renata Colombo Bonadio contributed equally to this paper

**Keywords:** advanced or recurrent endometrial cancer, immunotherapy, extracted individual patient data, meta-analysis

## Abstract

**Objective:**

To assess the impact of PD-1/PD-L1 inhibitors in first-line treatment of advanced or recurrent endometrial cancer (EC) through individual patient data (IPD) Meta-analysis, providing insights by integrated survival curves.

**Methods:**

We searched PubMed, Embase, Cochrane and meetings up to April 2024 for randomised phase II or III trials (randomised controlled trials) investigating immunotherapy plus chemotherapy for EC. IPD was reconstructed from Kaplan–Meier plots using WebPlotDigitizer and the R package IPDfromKM, and then combined.

**Results:**

NRG-GY018, RUBY, MITO END-3, AtTEnd/ENGOT-en7 and DUO-E were included. 2,436 patients were analysed for progression-free survival (PFS) and 2,317 for overall survival (OS). Among these, 621 patients had deficient DNA mismatch repair (dMMR) and 1,815 had the proficient disease (pMMR).

The IPD analysis highlighted the significant benefit of adding immunotherapy to chemotherapy in dMMR patients, with 3-year absolute gains of 36% in PFS (HR 0.36, 95% CI 0.28–0.45) and 28% in OS (HR 0.41, 95% CI 0.30–0.48).

For pMMR, a smaller benefit was observed in PFS, with a 3-year absolute gain of 6% (HR 0.78, 95% CI 0.69–0.88). Notably, a significant benefit occurred only with PD-1 inhibitors (PFS HR 0.66, 95% CI 0.55–0.79; OS: HR 0.78, 95% CI 0.62–0.96). No significant benefit was seen with PD-L1 inhibitors (PFS: 0.87, 95% CI 0.75–1.03; OS: HR: 0.93, 95% CI 0.75−1.16).

**Conclusion:**

This meta-analysis validated the benefit of adding immunotherapy to platinum-based chemotherapy with respect to PFS. dMMR patients gain advantages from the inclusion of either anti-PD-1 or anti-PD-L1 agents, whereas pMMR patients only experience this benefit when treated with anti-PD-1 agents.

## Introduction

Endometrial cancer (EC) stands as the most prevalent gynecologic malignancy in high-income nations, with its incidence on the rise due to the prevailing obesity epidemic [1]. While most cases are diagnosed early, affording an excellent prognosis with 5-year survival rates ranging from 74% to 91%, approximately 18% of cases present in advanced stages, leading to a significantly poorer prognosis, with a 5-year overall survival (OS) rate of 20%–25% when treated solely with chemotherapy [2, 3].

Until 2023, the standard first-line treatment of primary advanced or recurrent EC consisted of chemotherapy with carboplatin and paclitaxel [4]. Afterwards, treatment sequencing depended on the mismatch repair status. DNA mismatch repair deficient (dMMR) or microsatellite instability-high tumours, constituting 25% to 30% of cases [5], were typically treated with monotherapy using an immune checkpoint inhibitor, such as pembrolizumab or dostarlimab [6–8]. In contrast, pembrolizumab plus lenvatinib displayed efficacy in mismatch repair-proficient (pMMR) patients [9, 10]. 

Cytotoxic chemotherapy may exert immunomodulatory effects, including the interruption of immunosuppressive pathways and the augmentation of cytotoxic T-cell responses. Consequently, the combination of chemotherapy with immunotherapy could lead to synergistic effects within the tumour microenvironment. Besides that, clinical studies have indicated that this association can yield benefits, such as increased survival rates, across various cancer types [11–14].

Recent studies have evaluated the combination of immunotherapy with chemotherapy followed by immunotherapy maintenance as a first-line treatment, establishing it as a new standard of care. Four studies to date (NRG-GY 018, Ruby, Attend and Duo-E) provide robust evidence supporting the use of immunotherapy in first-line treatment for dMMR patients [15–18]. However, for patients with pMMR disease, the magnitude of progression-free survival (PFS) benefit is lower and the efficacy of PD-1 and PD-L1 inhibitors exhibits variability. While pembrolizumab and dostarlimab confer significant benefits in PFS for pMMR tumours, durvalumab shows a modest improvement, while atezolizumab and avelumab do not yield significant benefits [15–19].

To further elucidate these findings, we conducted a meta-analysis of extracted individual patient data (eIPD), evaluating the efficacy of anti-PD-1 and anti-PD-L1 agents in addition to first-line chemotherapy for advanced or recurrent EC. Additionally, the eIPD aimed to better illustrate the disease behaviour and the absolute gain of the immunotherapy agents over time by providing insights through the analysis of integrated survival curves.

## Methods

### Search strategy

We conducted a comprehensive search across PubMed, Embase and Cochrane databases for randomised controlled trials (RCTs) investigating the use of immunotherapy in first-line treatment of advanced or recurrent EC from inception until November 24th, 2023. Additionally, we reviewed abstracts presented in four oncology meetings (ASCO Annual Meeting, ESMO Congress, SGO annual meeting and IGCS annual meeting) until April 2024 to include new or updated results of trials that fulfilled our inclusion criteria. The detailed search query, included in Appendix, is structured around three key areas: (i) advanced or recurrent EC; (ii) immunotherapy (anti-PD1 or anti-PDL1 systemic therapies) and (iii) randomised clinical trials. Two investigators (RCB and MCG) independently evaluated retrieved abstracts, and discordances were solved through consensus with a third investigator (MMST). The most updated information from each trial was used if PFS or OS estimates were available. We followed the Preferred Reporting Items for Systematic Reviews and Meta-analyses (PRISMA) reporting guidelines [20]. The systematic review was registered in the PROSPERO database under the registration number CRD42024543401.

### eIPD meta-analysis

We reviewed publications and conference presentations for their Kaplan–Meier curves of PFS and OS. If more than one publication or presentation was available for an article, the most recent was included for analysis. Two independent investigators (MCG and MMST) eIPD of time-to-event outcomes built from KM curves using methods anteriorly described by Guyot *et al* [21] and Liu *et al* [22] with the R package ‘individual patient data (IPD)fromKM’. We assessed the quality of the IPD reconstruction curve by visual comparison of the reconstructed KM curves to the originally published curves and direct comparison of hazard ratios between curves. We examined the method’s reproducibility by comparing the curves of both investigators (MCG and MMST) (Supplementary Table 1).

From available eIPD data, we calculated survival median times and landmark survival probabilities with the Kaplan–Meier method and calculated hazard ratio with Cox proportional models. We estimated median OS and PFS between experimental (immunotherapy) and control arms for dMMR and pMMR patients in all trials, in trials of anti-PD-1 agents and in those of anti-PD-L1 agents.

All statistical analyses were performed in R version 4.2.2, with the packages’ ‘survival’ and ‘meta’. A *p*-value less than 0.05 was considered significant. Additional information on the risk of bias (RoB) evaluation, the eIPD and the trial-level meta-analysis are available in the Supplement.

## Results

### Study selection and descriptive analysis

**Figure 1.**
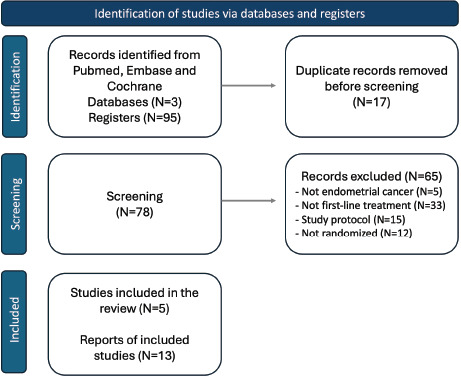
Flowchart of the systematic review and included publications.

Of 95 published studies, 5 articles with available PFS and/or OS data were included (PRISMA flow chart is shown in Figure 1): NRG-GY 018 [15], Ruby [16], Attend [17], Duo-E [18] and MITO-END 3 [19]. Trials detailed study population and treatments are described in Table 1.

### eIPD pooled analysis

**Figure 2.**
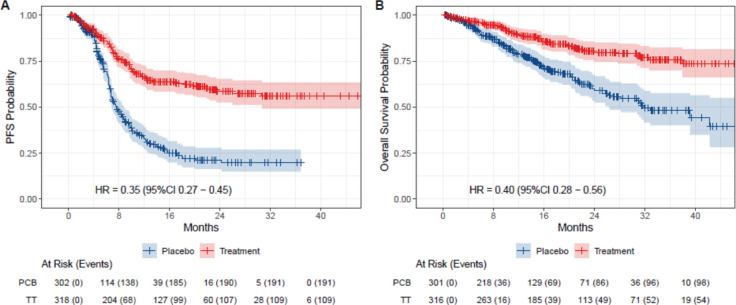
Kaplan-Meier plots of extracted IPD. Placebo arms are plotted in blue, while immunotherapy arms are plotted in red. (a): PFS for dMMR patients and (b): OS for dMMR patients.

In the eIPD meta-analysis of PFS, 2,436 patients were included, with 621 patients in the dMMR subgroup (318 in the immunotherapy arm versus 303 in the control arm) and 1,815 patients in pMMR subgroup (978 in the immunotherapy arm versus 837 patients in control arm). In the OS meta-analysis, 2,317 patients were included, with 560 patients in the dMMR group (290 in the immunotherapy arm and 270 in the control arm) and 1,757 in the pMMR group (947 in the immunotherapy arm and 810 in the control arm). Curve extraction was homogeneous for both evaluators, as shown in Supplementary Table 1. The pooled survival curves for PFS and OS are displayed in Figure 2 and the OS rates are estimated for each group over time, data of eIPD in 36 months according to MMR status and median survival are provided in Supplementary Tables 2–4.

The eIPD analysis of the five trials underscored the significant benefit of adding immunotherapy in dMMR patients, with 3-year absolute gains of 36% in PFS (HR 0.36, 95% CI 0.28–0.45) and 28% in OS (HR 0.41, 95% CI 0.30–0.48). In this group, survival curves showed early separation from first-line treatment initiation, reaching a plateau around 12 months, suggesting sustained benefit.

For the pMMR group, a smaller benefit was observed in PFS, with a 3-year absolute gain of 6% (HR 0.78, 95% CI 0.69–0.88). For OS, an absolute 3-year difference of 5% is observed, although the difference is not statistically significant, (HR 0.87, 95% CI 0.74–1.01). Notably, many PFS events occurred early in the pMMR curve, indicating numerous early progressors in both groups. A slight separation of curves began around 10 months, suggesting that a small subset of pMMR patients may benefit from adding immunotherapy to chemotherapy.

**Figure 3.**
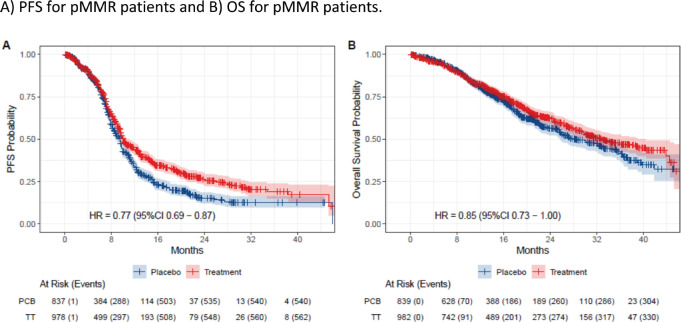
Kaplan-Meier plots of extracted IPD. Placebo arms are plotted in blue, while immunotherapy arms are plotted in red. (a): PFS for pMMR patients and (b): OS for pMMR patients.

**Figure 4.**
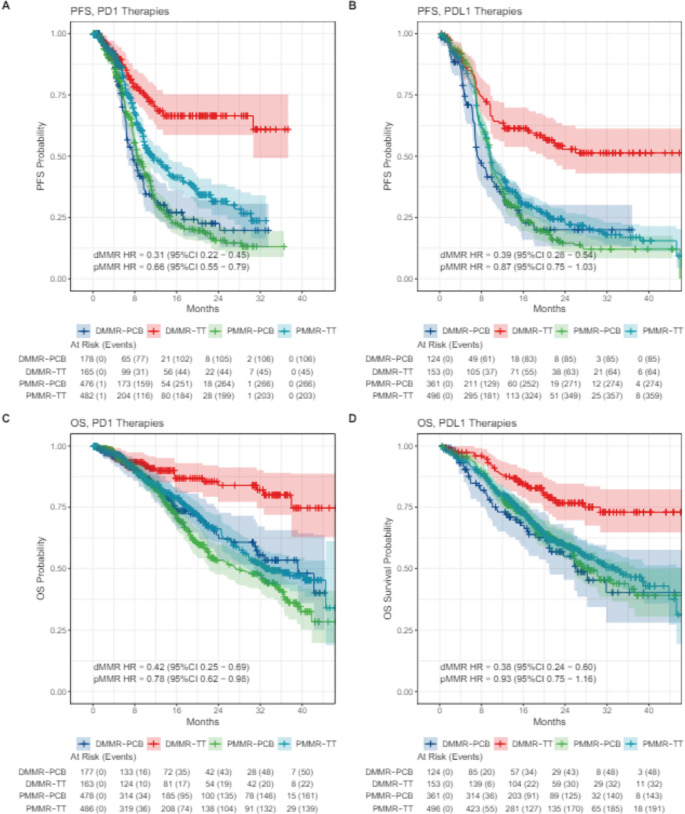
Kaplan-Meier plots of extracted IPD according to PD-1 and PD-L1 therapy and MMR subgroup. (a): PFS with anti-PD1 for dMMR and pMMR; (b): PFS for anti PD-L1 for dMMR and pMMR; (c) OS with anti PD-1 for dMMR and pMMR; (d) OS with anti PD-L1 for dMMR and pMMR.

In the eIPD analysis performed according to PD-1 (pembrolizumab and dostarlimab) and PD-L1 (atezolizumab, durvalumab and avelumab) inhibitors disparate results were observed (Figure 3). For dMMR patients, the magnitude of benefit was similar for PFS (PFS dMMR PD-1 HR 0.31, 95% CI 0.22−0.45; PFS dMMR PD-L1 HR 0.39 95% CI 0.28–0.54) and OS (OS dMMR PD-1 HR 0.42 95% CI 0.25−0.69; OS dMMR PD-L1 HR 0.38 95% CI 0.24−0.60) when using anti-PD-1 or anti-PD-L1 therapies. Notably, in the pMMR group, only the PD-1 inhibitors were associated with a statistically significant benefit in PFS (PFS PD-1 pMMR HR 0.66 95% CI 0.55−0.79). Moreover, an OS gain was also observed in the PD-1 inhibitors meta-analysis (OS PD-1 pMMR HR 0.78, 95% CI 0.62–0.96). On the other hand, the meta-analysis of PD-L1 inhibitors showed no significant benefit of these agents in PFS or OS (PFS PD-L1 pMMR HR 0.87 95% CI 0.75 − 1.03; OS PD-L1 pMMR HR 0.93, 95% CI 0.75−1.16) (Figure 4).

**Supplementary Figure 1.**
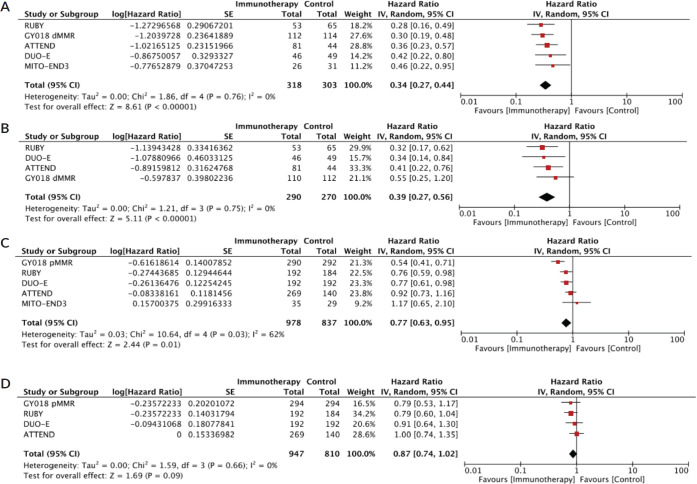
Graphical representation of the trial-level meta-analysis random effect models (a): PFS in the dMMR population, (b): OS in the dMMR population, (c): PFS in the pMMR population and (d): OS in the pMMR population with the respective forest plots and heterogeneity analysis results in the bottom-left.

**Supplementary Figure 2.**
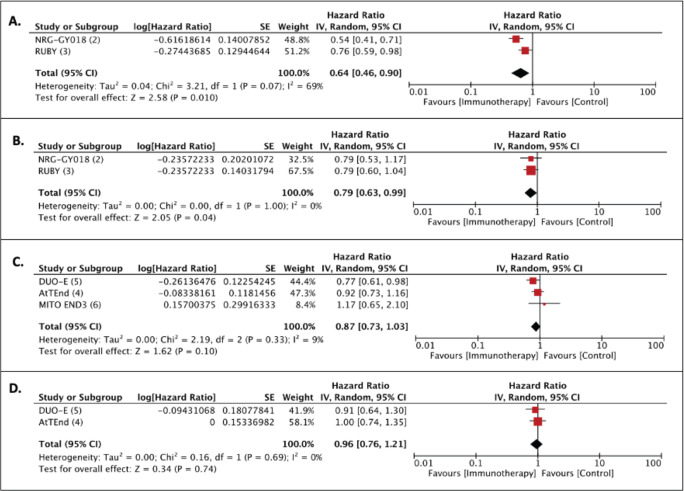
Trial level meta-analyses of first-line immune checkpoint inhibitor plus chemotherapy [Immunotherapy] versus chemotherapy [Control] for EC: (a): PFS with PD-1 inhibitors; (b): OS with PD-1 inhibitors; (c): Progression-free with PD-L1 inhibitors and (d): OS with PD-L1 inhibitors.

The trial-level meta-analyses are in line with the eIPD analyses and are provided in Supplementary Figures 1 and 2.

## Discussion

Since 2012, the standard first-line treatment for metastatic or recurrent EC has been a combination of carboplatin and paclitaxel. While this regimen has demonstrated high response rates, the duration of response has been limited. The year 2023 stood out for the treatment of advanced or recurrent EC as five clinical trials evaluating the addition of immunotherapy to standard chemotherapy in this setting were published or presented. Despite the differences in the selection of patients for those trials such as the treatment-interval after adjuvant chemotherapy, statistical design, histological subtypes permitted and duration of immunotherapy, data is consistent that dMMR is a strong predictive biomarker of response for the combination of chemotherapy with immune checkpoint inhibitors, independently of the mechanism of MMR loss, whether mutation or epigenetic alteration [23]. In our eIPD meta-analysis, the substantial and unprecedented gain of immunotherapy added to first-line chemotherapy for dMMR EC is reinforced, with a consistent benefit observed in PFS and OS with both anti-PD1 and anti-PD-L1 agents. On the other side, only a subset of unknown pMMR EC patients appears to benefit from immunotherapy. Moreover, this meta-analysis suggests a distinct efficacy of the agents in the pMMR group, with statistical significance observed only with PD-1 inhibitors.

Considering the remarkable response rates and PFS outcomes, in addition to the rationale of other tumour types that exhibit favourable outcomes in dMMR patients with single-agent immunotherapy, the necessity of chemotherapy in the first-line therapy for dMMR tumours remains uncertain [24]. The ENGOT-en9/LEAP-001 trial, evaluating pembrolizumab plus lenvatinib versus carboplatin plus paclitaxel in the first-line setting, showed an improvement in OS in favour of the interventional arm in a subgroup analysis of the dMMR patients (HR 0.57, 95% CI 0.36‒0.91) [25]. This result, although exploratory, suggests that regimens without chemotherapy might be an option in certain circumstances. Two ongoing RCTs will be able to further address this question, KEYNOTE-C93 (NCT05173987) and Domenica trial (NCT 05201547), comparing first-line pembrolizumab or dostarlimab, respectively, versus platinum-doublet chemotherapy in dMMR advanced or recurrent EC.

The duration of immunotherapy treatment for dMMR patients is also an open question. Survival curves showed early separation since first-line treatment initiation, reaching a plateau around 12 months, suggesting a sustainable benefit for those patients who remain in response upon the first year of treatment. However, the pivotal trials were designed with heterogeneous maintenance times of 2 or 3 years, or even indefinite maintenance, until progression or unacceptable toxicity [15-19].

In contrast to the dMMR group, only a small subset of patients with pMMR EC seems to benefit from the addition of immunotherapy, and our meta-analyses suggest the distinct efficacy of anti-PD1 and anti-PD-L1 agents in this setting. A statistically significant improvement in PFS was observed only with anti-PD1 agents, while the results for PD-L1 inhibitors were statistically not significant. Of note, the meta-analysis of anti-PD1 agents also showed a statistically significant OS benefit in the pMMR group. Since these agents exert their effects through distinct mechanisms, with singular particularities in the interaction with their targets, our results highlight that separate assessments of their activities in different scenarios might be informative. One possible reason for the enhanced effectiveness of anti-PD-1 is that anti-PD-1 antibodies can bind to PD-1 and simultaneously block its interaction with both ligands, PD-L1 and PD-L2. In contrast, while anti–PD-L1 antibodies inhibit the binding of PD-1 to PD-L1, they do not affect the interaction between PD-1 and PD-L2. This could allow tumours to evade the antitumour immune response via the PD-1/PD-L2 pathway when treated with anti-PD-L1 [26]. Additionally, the expression level of PD-L2 has been shown to be a significant predictor of survival benefit from immune checkpoint inhibitor treatment, regardless of the PD-L1 expression status [27, 28]. In line with our findings, a meta-analysis evaluating different cancer types suggested that PD-1 inhibitors were associated with a better objective response rate (21.6% versus 17.6%) and duration of response (11.26 versus 10.03 months) compared to PD-L1 inhibitors [29]. Different efficacies when comparing PD-1 and PD-L1 inhibitors were already described in other solid tumours [30].

Moreover, disparate outcomes between PD-1 and PD-L1 inhibitors are evident in biomarker-guided subgroup analyses. In a subanalysis of molecular characterization of RUBY part 1, PFS and OS results favoured the dostarlimab plus chemotherapy arm in the dMMR (HR 0.31, 95% CI 0.17–0.56) and in *TP53 mutated subgroups* (HR 0.55, 95% CI 0.30–0.99), but not in the no specific molecular profile (NSMP) subgroup (HR 0.77, 95% CI 0.55–1.07) [31]. On the other hand, in MITO END-3 analysis according to molecular profiling, it was found a statistically significant interaction of *TP53* mutation with avelumab treatment, suggesting that *TP53* mutations can be associated with resistance to immunotherapy (P interaction 0.003). The underlying reasons for these discrepant results, whether attributed to inherent differences in the activity of the anti-PD1 dostarlimab and the anti-PD-L1 avelumab or other factors such as trial design and study population, necessitate further investigation [32]. For instance, the imbalance in representation of the Asian population with almost 30% in DUO-E [18] versus approximately 3% in NRG-GY 018 [15] and Ruby [16] could contribute to these findings [33].

Notably, many PFS events occurred early in the pMMR eIPD curve, indicating numerous early progressors in both arms, but some patients seem to benefit from the combination of chemotherapy to immunotherapy with a separation of the curves around 10 months. This prompts important questions regarding strategies to distinguish these patients who benefit from those in whom treatment escalation or a different strategy is warranted. Furthermore, investigating additional biomarkers to differentiate subpopulations and the role of other therapeutic agents such as PARP inhibitors (PARPi) therapy are also subjects of interest [34, 35]. 

PARPi drive increased DNA damage in tumours with existing defects in DNA repair. This damage promotes immune priming through different molecular mechanisms, leading to the rationale of combining PARPi with chemotherapy and immunotherapy. To date, two phase III trials evaluated the benefit of adding PARPi, RUBY part 2 and DUO-E. Although both showed statistically positive results, they were not powered to compare the arm of PARPi plus chemotherapy and immunotherapy versus immunotherapy plus chemotherapy, the current standard of care. So, the question remains: who are the patients that benefit from the addition of PARPi. Reliable predictive biomarkers remain lacking, since the molecular characterization, PD-L1 expression, homologous recombination gene mutations and even *BRCA* mutational status have not proven sufficiently discriminatory in this context [18, 34, 35].

The main strengths of this meta-analysis are the eIPD that provide a plot that permits different interpretations of the curves, the consistent results with trial-level meta-analyses that were also performed and the subgroup analysis according to PD-1 versus PD-L1 inhibition, showing different outcomes for the pMMR populations. The weakness is that other meta-analysis on this theme were already published. Beyond that, the heterogeneity of the studies when it comes to the selection of patients, treatment interval after adjuvant chemotherapy, statistical design, histological subtypes included and duration of immunotherapy is also one limitation of the meta-analysis.

Other therapeutic strategies are also under evaluation. As immunotherapy migrates to the first-line scenario, antibody-drug conjugates might fill the gap left in the second line. Results from early-phase clinical trials are promising, both for anti-Trop2 and anti-HER2 agents. In the Destiny-PanTumour02, an overall response rate of 84.6% was seen for heavily pre-treated patients with HER2 3+ expressing EC [36, 37]. In addition, pMMR EC population is heterogenous and deserves further exploration of other biomarkers, once they present a complex molecular profile, with different targetable mutations, suggesting that new target agents can be investigated for advanced EC and some mutations can predict response, such as ARID1A (P interaction 0.01) and PTEN (P interaction 0.002) [32]. Target-based therapy with drugs affecting the PIK3CA and PTEN pathways is currently under investigation (Endomap trial NCT04486352). For now, Selinexor has shown promising activity as maintenance therapy following first-line carboplatin and paclitaxel in the prespecified subgroup analysis of patients with TP53 wild type/pMMR advanced disease increasing PFS from 4.9 months in the placebo group to 39.5 months, (HR 0.36; 95% CI 0.19–0.71) [38]. These findings from SIENDO trial are being further evaluated in the ongoing XPORT-EC-042 phase III trial (NCT05611931).

## Conclusion

In conclusion, while our study reinforces the substantial benefit of dMMR EC from both anti-PD1 and anti-PD-L1 agents in addition to first-line chemotherapy, it sheds light on the unmet needs of the pMMR group. Most patients with pMMR tumours exhibit early progression regardless of the immunotherapy agent and the identification of the subset who derives benefit from the therapy is still warranted. Moreover, the activity of the anti-PD-1 and anti-PD-L1 agents seems to diverge in the pMMR group, favouring the anti-PD-1 agents. Rigorous biomarker analyses and ongoing trial maturation are imperative to resolve these queries and delineate the precise role of immune checkpoint inhibitors in patients with pMMR EC.

## Conflicts of interest

FLN, MTC and MMST: declare no competing interest.

MCG: Speaker fees: Novartis, Knight Therapeutics; Financial support for educational programs and symposia: Pfizer, GSK.

RCB: Speaker fees and/or honoraria for consulting or advisory functions: Daiichi-Sankyo, Nestle Health Science, Zodiac, Gilead, MSD. Expert testimony: AstraZeneca, Ache, Nestle Health Science. Financial support for educational programs and symposia: AstraZeneca, Daiichi-Sankyo. Institutional - Research grant: Novartis, AstraZeneca;

MS: Speaker fees and/or honoraria for consulting or advisory functions: MSD, AstraZeneca, Libbs, GSK, Zodiac, Roche, Orentt, MDHealth, Sanofi, Gilead, Amgen, Sophia genetics.

## Funding

This research did not receive any funding.

## Author contributions

MS and RCB: Conceived the study.

MCG and MMST: Performed the extraction of individual patient data.

FLN, MTC and RCB: Performed the statistical analysis.

MCG: Wrote the initial version of the manuscript.

All authors: Reviewed the final manuscript.

## Figures and Tables

**Table 1.** Characteristics of the studies included in this present meta-analysis.

	NRG-GY018	RUBY	AtTEnd	DUO-E	MITO END-3
Phase	III	III	III	III	II
N	816 (dMMR: 225 | pMMR 591)	494 (dMMR: 118 | pMMR 376)	551 (dMMR: 125 | pMMR 426)	479* (dMMR: 95 | pMMR 384) *Excluding Durva + Ola	125 (dMMR: 57 | pMMR 68)
Primary end point	PFS in the cohort dMMR and pMMR	PFS dMMR → PFS ITT → OS ITT (hierarchically tested)	PFS dMMR → PFS ITT → OS ITT (hierarchically tested)	PFS ITT (Durva *vs.* control)	PFS ITT
Pts	Measurable disease (stage III- IVA) or stage IVB or recurrent EC	Primary advanced stage III-IV or first recurrent EC	Stage III-IV newly diagnosed or recurrent EC	Stage III-IV or recurrent EC	Stage III–IV or recurrent EC
First line	Paclitaxel + carboplatin + pembrolizumab/placebo for 6 cycles (q3w), followed by pembrolizumab/placebo for up to 14 cycles (q6w)	Paclitaxel + carboplatin + dostarlimab/placebo for 6 cycles (q3w), followed by dostarlimab/placebo for up to 3 years (q6w)	Paclitaxel + carboplatin + atezolizumab/placebo for 6 cycles (q3w), followed by atezolizumab/placebo until PD or unacceptable toxicity	Paclitaxel + carboplatin + durvalumab/placebo for 6 cycles (q3w), followed by durvalumab/placebo or durvalumab + olaparib until PD or unacceptable toxicity (q4w)	Paclitaxel + carboplatin + avelumab/placebo for 6 cycles (q3w), followed by avelumab/placebo until PD or unacceptable toxicity (q2w)
Median FUP	12 months in the dMMR cohort and 7.9 months in the pMMR	24.8 months in the dMMR population and 25.4 months in the ITT	26.2 months in the dMMR population and 28.3 months in the ITT	16.4 months in the control arm and 17.1 months in Durva arm	23.3 months for both arms
PFS ITT	NR	HR 0.64 95% CI: 0.51-0.80	HR 0.74 95% CI: 0.61-0.91	HR 0.71 95% CI: 0.57–0.89	HR 0.78 95% CI: 0.65–0.93
PFS dMMR	HR 0.30 95% CI: 0.19-0.48	HR 0.28 95% CI: 0.16-0.50	HR 0.36 95% CI: 0.23-0.57	HR 0.42 95% CI: 0.22–0.80	HR 0.46 95% CI: 0.22-0.94
PFS pMMR	HR 0.54 95% CI: 0.41-0.71	HR 0.76 95% CI: 0.59-0.98	HR 0.92 95% CI: 0.73-1.16	HR 0.77 95% CI: 0.60–0.97	HR 1.17 95% CI: 0.65-2.10
OS ITT	NR	HR 0.69 95% CI: 0.54-0.89	HR 0.82 95% CI: 0.63-1.07	HR 0.77 95% CI: 0.56–1.07	HR 1.13 95% CI: 0.62–2.07
OS dMMR	HR 0.55 95% CI: 0.25 - 1.19	HR 0.32 95% CI: 0.17-0.63	HR 0.41 95% CI: 0.22-0.76)	HR 0.34 95% CI: 0.13-0.79	NR
OS pMMR	HR 0.79 95% CI: 0.53 - 1.17	HR 0.79 95% CI: 0.60-1.04	HR 1.00 95% CI: 0.74-1.35	HR 0.91 95% CI: 0.64-1.30	NR

Abbreviations: N = Number of patients, PFS = progression-free survival, OS = Overall survival, ITT = Intention to treat, pMMR = mismatch repair–proficient, dMMR = mismatch repair–deficient, pts = patients, q2w = every 2 weeks, q3w = every 3 weeks, q4w = every 4 weeks; q6w = every 6 weeks; w = weeks, HR = Hazard ratio, 95% CI = 95% Confidence interval, FUP = follow-up; NR = not reported; placebo = plb; Durva = Durvalumab; Ola = Olaparib; PD = progressive disease

**Supplementary Table 2.**
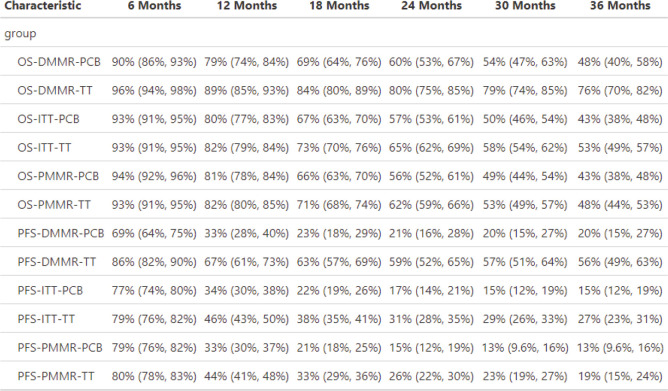
OS rates estimated for each group as time goes by.

**Supplementary Table 3.**
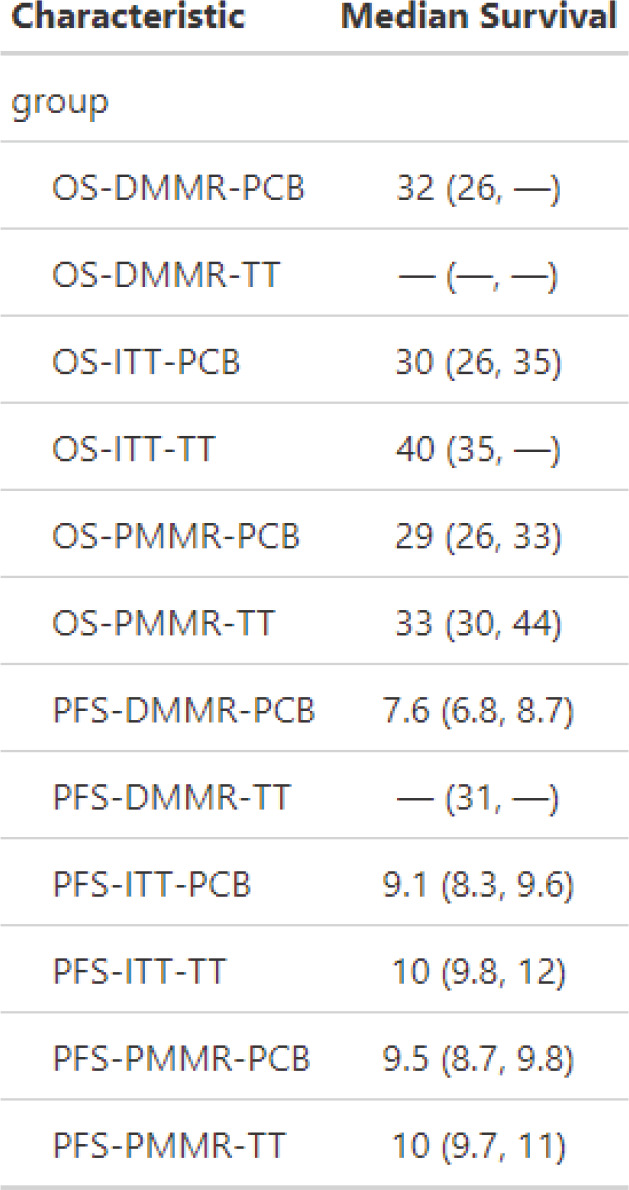
Median survivals.

## Supplement

### Material and methods

#### Risk of bias

RoB was evaluated with the Cochrane RoB tool for randomised controlled trials by one of the investigators (MTC). In general, all RCTs were evaluated as having a low RoB.

#### eIPD meta-analysis - proportional hazards and restricted mean survival times (RMST)

We investigated the proportional hazards assumption by inspecting Schoenfield residuals. We evaluated this assumption for each group separately (dMMR and pMMR) because we expected different curves by MMR status. When proportional hazards assumption was violated, the difference of RMST-D, a non-parametric test to compare the area under the Kaplan–Meier curves of each arm, was calculated14. To avoid bias due to follow-up difference, RMST-D time threshold (τ) was defined as the shortest upper boundary of follow-up times between trials. Additionally, we investigated the presence of informative censoring with the reverse Kaplan–Meier method where actual events are depicted as censored, and each censor point is treated as an event. This method provides the hazards of being censored over-time and has been previously validated13. We opted to investigate informative censoring only in the all-comers population as it is not expected that MMR status would influence informative censoring.

#### Trial-level meta-analysis

We extracted point and corresponding confidence interval limits estimates of EFS and OS hazard-ratio from each trial and calculated the pooled hazard ratio and corresponding 95% confidence interval using both fixed- and random-effects models. Heterogeneity among studies were assessed using τ2, I2 and Cochran’s *Q* statistics.

### Results

#### Proportional hazards assumption and informative censoring

Inspection of Schoenfeld residuals has found evidence of proportional hazards violation for PFS curves from Ruby dMMR, DUO-E pMMR and OS curves from MITO pMMR. RMST differences in these studies pointed in the same direction of original effect and significance (Supplementary Table 4). We have not found evidence of informative censoring in analysed trials (Supplementary Table 5).

#### Trial-level meta-analysis

The trial-level meta-analysis for PFS included data from NRG-GY018, 15 RUBY, 16 MITO- END 3, 17 DUO-E 18 and Attend. 19 The analysis was performed according to the MMR status. For dMMR, it showed low heterogeneity (PFS dMMR: I2 = 0%, τ2 = 0.00, *Q p*-value = 0.76) and, in line with findings from IPD analysis, showed PFS benefit for the immunotherapy group compared to control in random effects model (HR 0.34, 95% CI 0.27–0.44). On the other hand, in the pMMR subgroup, there was a higher heterogeneity (PFS pMMR: *I*^2^ = 62%, τ2= 0.03, *Q p*-value = 0.03). The results also favoured the intervention arm in pMMR subgroup: random effects model (HR 0.77, 95% CI 0.63–0.95)

The OS analysis was also done depending on the MMR status. For the dMMR, an important benefit was observed (HR 0.39, 95% CI 0.27–0.56). However, the OS results for pMMR were not statistically favourable (HR 0.87, 95% CI 0.74–1.02). The trial-level meta-analyses, along with heterogeneity measures.

**Supplementary Table 1.** Curve extraction according to author 1 and 2.

Analysis	Study HR	Author 1- HR	Author 2- HR
DUO E ITT OS	0.77	0.76	0.79
DUO E ITT PFS	0.71	0.68	0.68
DUO E PMMR OS	0.91	0.93	0.92
DUO E DMMR OS	0.34	0.30	0.34
DUO E DMMR PFS	0.42	0.43	0.43
DUO E PMMR PFS	0.77	0.79	0.77
ATTEND DMMR OS	0.41	0.41	0.41
ATTEND DMMR PFS	0.36	0.37	0.38
ATTEND ITT OS	0.82	0.81	0.83
ATTEND ITT PFS	0.74	0.75	0.75
ATTEND PMMR OS	1.0	1.00	1.01
ATTEND PMMR PFS	0.92	0.92	0.92
MITO DMMR OS	NR	0.36	0.33
MITO DMMR PFS	0.46	0.37	0.39
MITO ITT OS	1.13	1.00	1.01
MITO ITT PFS	0.78	0.75	0.74
MITO PMMR OS	NR	0.54	0.53
MITO PMMR PFS	1.17	1.18	1.20
NRG DMMR PFS	0.30	0.30	0.32
NRG PMMR PFS	0.54	0.56	0.56
RUBY DMMR OS	0.32	0.35	0.34
RUBY DMMR PFS	0.28	0.33	0,36
RUBY ITT OS	0.69	0.65	0.69
RUBY ITT PFS	0.64	0.63	0.62
RUBY PMMR OS	0.79	0.78	0.78
RUBY PMMR PFS	0.76	0.75	0.76

Abbreviations: pMMR = proficient mismatch repair, dMMR = deficient mismatch repair, HR = Hazard ratio, PFS = progression-free survival, OS = Overall survival, NR: Not reported

**Supplementary Table 4.** Proportional hazards.

Study	Population	Outcome	Treat. Effects HR	PH schoenfeld residuals test *p*-value	RMST difference
RUBY	DMMR	OS	0.34 (0.18–0.66)	0.2102	
RUBY	PMMR	OS	0.78 (0.59–1.03)	0.8534	
RUBY	DMMR	PFS	0.33 (0.19–0.56)	**0.0240**	10.618 (6.194–15.043)
RUBY	PMMR	PFS	0.76 (0.59–0.98)	0.7479	
NGR	DMMR	PFS	0.30 (0.19–0.48)	0.6850	
NGR	PMMR	PFS	0.56 (0.43–0.73)	0.3477	
NGR	DMMR	OS	0.58 (0.26–1.29)	0.7602	
NGR	PMMR	OS	0.78 (0.53–1.15)	0.4321	
MITO	PMMR	PFS	1.18 (0.67–2.07)	0.5684	
MITO	DMMR	PFS	0.39 (0.19–0.78)	0.6772	
MITO	PMMR	OS	0.54 (0.23–1.29)	**0.0412**	4.549 (−2.564–11.662)
MITO	DMMR	OS	0.36 (0.13–1.02)	0.5480	
DUOE	DMMR	PFS	0.43 (0.22–0.82)	0.8668	
DUOE	PMMR	PFS	0.79 (0.62–1.00)	**0.0173**	2.097 (0.279–3.916)
DUOE	DMMR	OS	0.34 (0.14–0.81)	0.7513	
DUOE	PMMR	OS	0.92 (0.64–1.31)	0.7995	
ATTEND	DMMR	PFS	0.37 (0.23–0.59)	0.4594	
ATTEND	PMMR	PFS	0.92 (0.73–1.16)	0.1418	
ATTEND	DMMR	OS	0.41 (0.21–0.79)	0.4010	
ATTEND	PMMR	OS	1.00 (0.74–1.35)	0.1127	

Bold values:* p* < 0.05

**Supplementary Table 5.** Informative censoring analysis (Reverse Kaplan-Meier method).

Study	Population	Outcome	HR (Reverse KM)	*p*-val
RUBY	ITT	OS	1.00 (0.78–1.29)	0.9992
RUBY	ITT	PFS	1.11 (0.83–1.50)	0.4899
MITO	ITT	PFS	1.11 (0.56–2.19)	0.7651
MITO	ITT	OS	1.04 (0.67–1.62)	0.8882
DUOE	ITT	PFS	0.88 (0.63–1.21)	0.3800
DUOE	ITT	OS	1.03 (0.83–1.28)	0.8003
ATTEND	ITT	PFS	0.75 (0.52–1.07)	0.1102
ATTEND	ITT	OS	0.95 (0.75–1.21)	0.6613
